# Clinical Society of Maryland

**Published:** 1892-07

**Authors:** W. T. Watson

**Affiliations:** Secretary; 1519 N. Broadway, Baltimore


### *SOCIETY REPORTS.*

### CLINICAL SOCIETY OF MARYLAND.

BALTIMORE, MD., May 6, 1892.

DR. H. A. KELLEY spoke on

INJURIES TO THE VAGINAL OUTLET, illustrating his remarks by many beautifully prepared photographs thrown upon a screen by a stereopticon. The photographs were all taken from cases under Dr. Kelly's care both in Philadelphia and in the Johns Hopkins Hospital, Baltimore, and illustrated in the operating room, normal vaginal outlets, injured outlets of different kinds, and the various steps in the operation for restoration.

DR. KELLEY said, in part: Injuries to the vaginal outlet are of three sorts: First, involving the *external anterior part of the perineum*, that is the tissue from the fourchette backwards toward the anus and upward toward the vagina. This is a superficial tear of no serious moment. The second form is the *complete tear* involving the whole of the recto-vaginal septum. Here the sphincter is ruptured, and as a consequence the patient is unable to control her bowels or gases. The fact that a prolapsus is so rarely associated with this condition shows that the perineal body is not the supporting structure of the pelvic organs. The third form is the *internal tear*, that is where the rupture lies in the right or left side of the vagina or both, and can only be seen on drawing apart the labia and lifting up the anterior wall with the speculum. This tear is concealed and is rarely seen. It is, however, from its frequency and its effect the most important of all, for the result of such an injury is relaxation of the vaginal outlet. The rupture in the sulci extends down into the tissues separating the levator ani from its rectal attachments; the muscle is, therefore, no longer able to hold the rectum up under the pubic arch; the anus drops backwards, the vaginal walls roll out, and often without any external injury whatever, or even with an external perineum larger than normal from the overstretching. Extensive eversion of the vagina with decensus of the uterus is one of the sequences of the inside tear.

The *operation* for such injury is resection of the relaxation. There is no advantage in an operation that does not sacrifice any of the tissue; the best treatment is to take out of the relaxation just enough tissue to bring it back again to the normal size. It is not proper to confine this resection of the vaginal outlet to the exterior alone. The injury is more on the inside, and for this reason the operation is also made to extend up the vagina by making the denudation or resection in both the sulci and across the lower anterior face of the posterior vaginal wall. Two triangular areas of denudation point up both sulci ; in these the tissues on either side are loosely approximated by means of a single silk-worm-gut suture to each sulcus. This is the tension suture. A number of cat-gut sutures are then able to do the work of approximation above this. (Cat-gut must never be used where there is much tension.) The lower part of the denudation is brought together by silk-worm-gut sutures passed in a transverse direction. An outlet thus restored appears perfectly normal or even virginal. If you were to examine such an outlet a year or two after operation, you would say that the woman had an intact perineum.

DR. J. EDWIN MICHAEL agreed with Dr. Kelley that the essence of this whole matter is the amount of damage inflicted upon the sphincter ani muscle. Sometimes a long perineum is a better indication of internal trouble than a torn perineum. The operation referred to by Dr. Kelley in which no tissue is lost can hardly compare with the operation which Dr. Kelley practices ; it cannot take up the broken ends of the sphincter and tuck them up under the pubes, and that is what is wanted in these cases.

We are apt in these cases to do too much and to make the vaginal outlet too narrow. The point on the labium at which the normal cleft ceases is comparatively easily determined and this enables us to give the proper size to the external opening ; but in our internal denudations we are inclined to do too much and produce too great narrowing.

I recently attended a patient in labor who had previously had a tear repaired by the method which has been explained by Dr. Kelley. The new-made perineum withstood the trial of a second labor and is now about as good as before the labor occurred.

We ought to be thankful to Dr. Kelley for taking advantage of the circumstances with which he is surrounded and illustrating the subject in this vivid way.

J. H. BRANHAM : The tear of the levator ani muscle may sometimes be extensive without any laceration of the mucous membrane ; and on the other hand the mucous membrane may be extensively lacerated with very little laceration of the muscles. Nature of course attempts to repair these damages, but the general practitioner, in allowing his patient to move about and sit up too soon, thus allowing pressure from above on the torn muscles, interferes with the efforts of nature. The condition should be recognized and the patient kept long enough in bed to permit of thorough repair.

DR. HENRY FRIEDENWALD read a paper on

CRANIAL DEFORMITY AND OPTIC NERVE ATROPHY.

DR. J. G. PRESTON thought the nerve atrophy was due to certain inflammatory conditions of the meninges and special pressure, and not due to general intracranial pressure.

The operation of linear craniectomy has not been a success thus far. The operation gives an opportunity for brain expansion, but very little. The damage has usually been done before the operation is undertaken.

DR. FRIEDENWALD: In cases of tumors of the brain, which are found far away from the anterior part of the brain, optic neuritis follows, and it is very hard to account for it except on the theory of increased intracranial pressure, and my suggestion was that in cases of marked cranial deformity, we are probably likely to have at some period a time when the intracranial pressure reaches the same height as it would in the case of intracranial tumor.

DR. I. E. ATKINSON narrated a case of Rapidly Growing Intra-Thoracic Tumor, ending in death by suffocation in six weeks after first consulting his physician. The tumor, an aneurism of the innominate artery, was exhibited by Dr. K. B. Batchelor.

W. T. WATSON, Secretary.

1519 *N. Broadway, Baltimore.*

---

AT a special meeting of the Board of Trustees of the Medico-Chirurgical College, of Philadelphia, Dr. W. Frank Haehnlen, Demonstrator of Obstetrics at the University of Pennsylvania, was elected Professor of Obstetrics; Dr. W. Easterly Ashton, Lecturer on Gynecology at Jefferson Medical College, Professor of Gynecology; Dr. Chas. M. Seltzer, Professor of Hygiene; Dr. H. H. Boom, Adjunct Professor of Chemistry; Dr. B. M. Shimwell, Adjunct Professor of Operative Surgery.

